# Small RNAs and Gene Network in a Durable Disease Resistance Gene—Mediated Defense Responses in Rice

**DOI:** 10.1371/journal.pone.0137360

**Published:** 2015-09-03

**Authors:** Hanming Hong, Yanyan Liu, Haitao Zhang, Jinghua Xiao, Xianghua Li, Shiping Wang

**Affiliations:** National Key Laboratory of Crop Genetic Improvement, National Center of Plant Gene Research (Wuhan), Huazhong Agricultural University, Wuhan 430070, China; Ohio State University, UNITED STATES

## Abstract

Accumulating data have suggested that small RNAs (sRNAs) have important functions in plant responses to pathogen invasion. However, it is largely unknown whether and how sRNAs are involved in the regulation of rice responses to the invasion of *Xanthomonas oryzae* pv. *oryzae* (*Xoo*), which causes bacterial blight, the most devastating bacterial disease of rice worldwide. We performed simultaneous genome-wide analyses of the expression of sRNAs and genes during early defense responses of rice to *Xoo* mediated by a major disease resistance gene, *Xa3/Xa26*, which confers durable and race-specific qualitative resistance. A large number of sRNAs and genes showed differential expression in *Xa3/Xa26*-mediated resistance. These differentially expressed sRNAs include known microRNAs (miRNAs), unreported miRNAs, and small interfering RNAs. The candidate genes, with expression that was negatively correlated with the expression of sRNAs, were identified, indicating that these genes may be regulated by sRNAs in disease resistance in rice. These results provide a new perspective regarding the putative roles of sRNA candidates and their putative target genes in durable disease resistance in rice.

## Introduction

Rice is the main staple food for a large part of the world. Rice diseases cause great global loss of yield every year [1]. Using intrinsic genetic defense resources in rice for improvement in disease resistance is an important strategy for sustainable rice production [2]. Breeding rice with the features of broad-spectrum (against two or more types of pathogen species or the majority of strains or isolates of the same pathogen species) and durable (remaining effective during its prolonged and widespread use in an environment favorable to disease spread) disease resistance is one of the principal goals of rice improvement [3]. However, the effective use of defense resources for rice breeding is dependent on our understanding of rice defense mechanisms that still remain to be elucidated.

According to the strength and the speed of plant response to pathogen infection, rice resistance to pathogens can be genetically classified into two classes: qualitative or complete resistance and quantitative or partial resistance (as described in detail previously [3, 4]). Qualitative resistance is mediated by major disease resistance (*MR*) gene(s) and is a gene-for-gene type resistance [4]. The molecular basis of qualitative resistance in some rice—pathogen pathosystems can be explained using the effector-triggered immunity (ETI) model or the pathogen-associated molecular pattern (PAMP)-triggered immunity (PTI) model (or microbe-associated molecular pattern [MAMP] model), or both [4, 5]. Quantitative resistance is mediated by quantitative trait loci (QTLs) and can frequently be a broad-spectrum and durable resistance [3, 4]. Several disease resistance QTLs in rice have been characterized, some of which function in *MR* gene—initiated defense transduction pathways, suggesting that resistance QTLs may be important components in *MR* gene—mediated defense signaling [3, 6–8].

Accumulating data have revealed that host endogenous-generated small RNAs (sRNAs), including microRNAs (miRNAs) and small interfering RNAs (siRNAs), play roles in plant—pathogen interactions [9]. These sRNAs either promote or repress defense responses by suppressing host gene expression at the mRNA or DNA level [10, 11]. A set of miRNAs was identified to contribute to the regulation of PTI or ETI in Arabidopsis [12–17]. Recent studies have revealed that miRNAs are also involved in rice response to the invasion of the fugal pathogen *Magnaporthe oryzae*. Overexpressing osa-miR7695 promotes rice resistance to *M*. *oryzae* by suppressing a metal transporter gene [18]. Overexpressing miR160a or miR398b in rice enhanced resistance to *M*. *oryzae*, which was associated with increased H_2_O_2_ accumulation at the infection site and upregulation of the expression of defense-related genes [19]. In addition to siRNAs, miRNAs may also be involved in rice resistance to viruses [20]. However, the complete roles of sRNA in plant defense responses to pathogen invasion, especially in rice—bacterium interactions, are far from understood.

Bacterial blight caused by *Xanthomonas oryzae* pv. *oryzae* (*Xoo*) is the most devastating bacterial disease of rice worldwide [21]. However, based on our knowledge, it is unknown whether sRNAs are involved in the rice response to *Xoo*. The *MR* gene *Xa3/Xa26*, which encodes a leucine-rich repeat kinase-like protein, confers broad-spectrum but race-specific resistance to *Xoo* [22, 23]. *Xa3/Xa26* has played an important role in rice production. *Xa3/Xa26*-carrying rice cultivars have been widely planted in China for a long period of time [21]. The *Xa3/Xa26* alleles *Xa3/Xa26-2* and *Xa3/Xa26-3*, which encode proteins with high sequence similarity to the Xa3/Xa26 protein, from wild rice species (BB and BBCC genomes) that separated from the ancestor (AA genome) of cultivated rice approximately 7.5 million years ago mediate the same spectrum of resistance to *Xoo* as *Xa3/Xa26* [24]. These features indicate that the *Xa3/Xa26* locus confers durable resistance. Furthermore, six genes (*OsDR8*, *OsDR10*, *GH3-8*, *WRKY13*, *WRKY45*, and *C3H12*), some of which contribute to resistance QTLs, that function in *Xa3/Xa26*-initiated defense pathways have been identified, and these genes are either positively or negatively involved in rice resistance against *Xoo* [7, 8, 25–31]. At least three of the six genes can also mediate rice resistance to other pathogen species in addition to *Xoo*, suggesting that activation of *Xa3/Xa26* can also lead to broad-spectrum disease resistance to multiple pathogen species. However, it is unknown whether sRNAs are involved in *Xa3/Xa26*-mediated resistance.

To better understand the defense mechanisms conferred by *Xa3/Xa26*, we profiled the genome-wide expression of sRNAs and genes in the rice response to *Xoo* using Solexa high-throughput sequencing technology and Affymetrix GeneChip. This analysis has revealed a large number of differentially expressed sRNAs and defense-responsive genes in rice—*Xoo* interactions, suggesting that sRNAs may be involved in *Xa3/Xa26*–mediated defense signaling. These transcriptomes provide a comprehensive perspective on the putative roles of sRNAs in rice resistance against *Xoo*.

## Materials and Methods

### Rice materials and growth conditions

Japonica rice (*Oryza sativa* ssp. *japonica*) variety Mudanjiang 8 (MDJ8) is the wild-type and is susceptible to *Xoo*. Transgenic rice line Rb49 carries the *MR* gene *Xa3/Xa26*, which is driven by its native promoter with the genetic background of MDJ8, and this line is resistant to certain strains (including strain PXO61) of *Xoo* [22, 32]. Rice plants were grown in the field under nonstressed conditions during the rice-growing season.

### Plant treatment

Plants were inoculated with the *Xoo* strain PXO61 at the four-leaf to five-leaf stage by the leaf-clipping method [33], and disease was scored by measuring the lesion area percentage (lesion length/leaf length) at 10 days after inoculation. Control rice plants were inoculated with water (mock inoculation). Samples were collected before inoculation (ck) and at 2, 4, and 24 hours after PXO61 or mock inoculation from Rb49 and MDJ8. Leaf fragments approximately 2 cm in length that were immediately next to the inoculation site were collected. The leaf samples for each treatment of each replication involved a pool of 15 to 20 plants. The frozen samples were ground into powder in liquid nitrogen and each sample was divided into two parts, one for sRNA sequencing and another for gene chip analysis. The samples were kept in TRIZOL reagent (Invitrogen, Gaithersburg, MD) at −70°C.

### sRNA analysis

The sRNA isolation, library construction, and sequencing were performed by CapitalBio (Beijing, China). In brief, total RNA was extracted with TRIZOL reagent according to the manufacturer’s instructions. The quality of total RNA was examined first. Total RNA for each library was ligated first to a 3′ RNA adaptor and then to a 5′ RNA adaptor. A reverse-transcription (RT) reaction was followed by polymerase chain reaction (PCR) to obtain sufficient products. The PCR products with lengths between 145 and 160 nucleotides (nt) were isolated by 6% polyacrylamide gel electrophoresis and then sequenced using an Illumina Genome Analyzer IIx.

The raw deep-sequencing data were pre-processed by removing low-quality reads and reads smaller than 18 nt, by removing contamination formed by adaptor—adaptor ligation, and by trimming adaptor sequences. The clean reads ranging from 18 to 30 nt were mapped to the rice genome based on the Rice Genome Annotation Project (RGAP; http://rice.plantbiology.msu.edu) version 7.0 using the Bowtie program [34].

The classification of miRNAs was performed according to processes reported previously [35]. The known miRNAs were determined by searching the miRBase (http://www.mirbase.org/, release 21). Some sRNAs in the miRBase are from transposable elements. To avoid confusion, we still classified these sRNAs into the miRNA group in this study. To identify putative new miRNA, the sequences, which completely matched the genomic sequences, were first classified as tRNA, rRNA, small nuclear RNA (snRNA), small nucleolar RNA (snoRNA), or mRNA by searching the National Center for Biotechnology Information (NCBI; http://www.ncbi.nlm.nih.gov) nucleotide database and the RNA family database (Rfam; http://rfam.xfam.org, release 11) [36]. The rest sequences were classified as trans-acting siRNA 3 (TAS3) genes by searching tasiRNAdb (http://bioinfo.jit.edu.cn/tasiRNADatabase/) [37] or as repeat sequence by searching the Oryza Repeat Database (http://rice.plantbiology.msu.edu) [38]. The further rest sequences were classified as pre-miRNA by searching the miRBase or as rice intron by searching the RGAP. The sRNAs, whose sequences matched pre-miRNAs, but not the known miRNAs, were considered new miRNAs. Then, the unannotated reads were used for prediction of new miRNAs according to a pipeline described previously [35]. In brief, sRNA generated from a precusor sequence, which did not belong to the aforementioned sequences and was predicted to form a hairpin-like structure, was considered to be novel.

To avoid false identification, only the sequencing reads having the structure characteristic of siRNA, unique position on the reference genome, and perfect match with the reference genome were used for siRNA analysis [39, 40]. The phasing siRNA analysis was performed as described previously [41].

The total clean reads of each sample were normalized into reads per million (RPM) for the expression of miRNAs and siRNAs to determine differentially expressed sRNAs. Differentially expressed sRNAs were determined by comparison between pathogen-inoculated and mock-treated samples or between samples from MDJ8 and Rb49 according to a standard reported previously [42]. Based on this standard, if the sRNA fold-change is more than two or if the fold-change is less than 0.5, the *P* value smaller than 0.05 but larger than 0.01 is considered a difference and the *P* value smaller than 0.01 is considered a significant difference. The *P* value was determined by hypergeometric distribution. The potential target genes of sRNA were predicted using the psRNATarget program (http://plantgrn.noble.org/psRNATarget/) with default parameters [43].

### Gene chip analysis

Each treatment had two biological replicates. Affymetrix GeneChip Rice Genome Arrays (http://www.affymetrix.com/catalog/131497/AFFY/Rice+Genome+Array#1_1) were used for genome-wide gene expression analysis. This chip contains 57,381 probe sets representing 51,279 transcripts; it was designed based on the predicted 59,712 genes. RNA purification, quality assessment, and labeling as well as array hybridization were conducted at CapitalBio according to the manufacturer’s protocol (Affymetrix GeneChip Expression Analysis Technical Manual). The scanned images were quantified using AGCC (Affymetrix GeneChip Command Console) software with the CEL files. Then, we used Bioconductor packages (http://www.bioconductor.org) for the following statistical analyses. The CEL files were imported into the R environment and were background-corrected and normalized using the robust multi-array average (RMA) algorithm in the affy package [44]. The probe sets called "Present", which were determined by the mas5 algorithm in the affy package, in at least one rice sample were considered as expressed and were used in further analyses. The limma package, then, was used for the analysis of differentially expressed probe sets between samples [45]. The resulting *P*-value was adjusted by the method of Benjamini and Hochberg to control the false discovery rate [45]. Criteria for the significantly differentially expressed probe set were q ≤ 0.05 and fold-change of two or more.

### Classification of genes

To classify the genes that were putative targets of sRNAs or differentially expressed in gene chip analysis, the Gene Ontology (GO) [46] term was used to perform the classification analysis. The AgriGO program was used for this analysis for the terms "biological process", "molecular function", and "cellular component" with default parameters [47]. RGAP version 7.0 non-TE was selected as the background for selecting references.

### Analysis of the correlation of expression of sRNAs and their target candidates

The web server psRNATarget was used to predict the potential target genes of differentially expressed miRNAs, and RGAP version 7.0 was used for the target search [48]. Pearson’s correlation coefficient (PCC) was used to evaluate the correlation between miRNAs and their targets [35].

### Quantitative RT-PCR

The same total RNA samples used for sRNA sequencing and gene chip analysis were used for quantitative RT-PCR (qRT-PCR) analyses. The sRNA expression analysis was performed according to a reported method using sRNA-specific stem-loop RT primers and quantitative PCR primers (S1 Table) [49]. The expression level of rice snRNA U6 was used to standardize the RNA sample for each qRT-PCR. For gene expression analysis, qPCR was performed as described previously using gene-specific primers listed in S2 Table [26]. The expression level of the rice actin gene was used to standardize the RNA sample for each qRT-PCR. The expression level of each sRNA or gene in treated rice plants was determined relative to that in untreated rice plants [26].

## Results and Discussion

The seedlings of resistant rice line Rb49 and susceptible rice line MDJ8 were inoculated with *Xoo* strain PXO61 or water (mock inoculation as control). The resistant reaction of Rb49 (lesion area 8.1 ± 2.68%) and the susceptible reaction of MDJ8 (lesion area 47.1 ± 7.15%) were confirmed at 10 days after inoculation, indicating the reliability of the inoculated samples for this study. A preliminary experiment was conducted to check the reliability of the sequencing and sample preparation. A sample collected from Rb49 at 4 hours after mock inoculation (Rb49-4h-mock) was sequenced twice; the correlation of miRNA abundance between the two technical duplicates was 0.971 (S1A Fig). The MDJ8-ck sample had two biological replicates; the correlation of miRNA abundance between the two replicates was 0.978 (S1B Fig). The correlation between the two biological replicates of each treatment for gene chip analyses ranged from 0.993 to 0.998 (S3 Table). These results suggest that the sequencing data and gene chip data are reliable.

### There is a large numbers of rice line—specific sequencing reads

**Fig 1:**
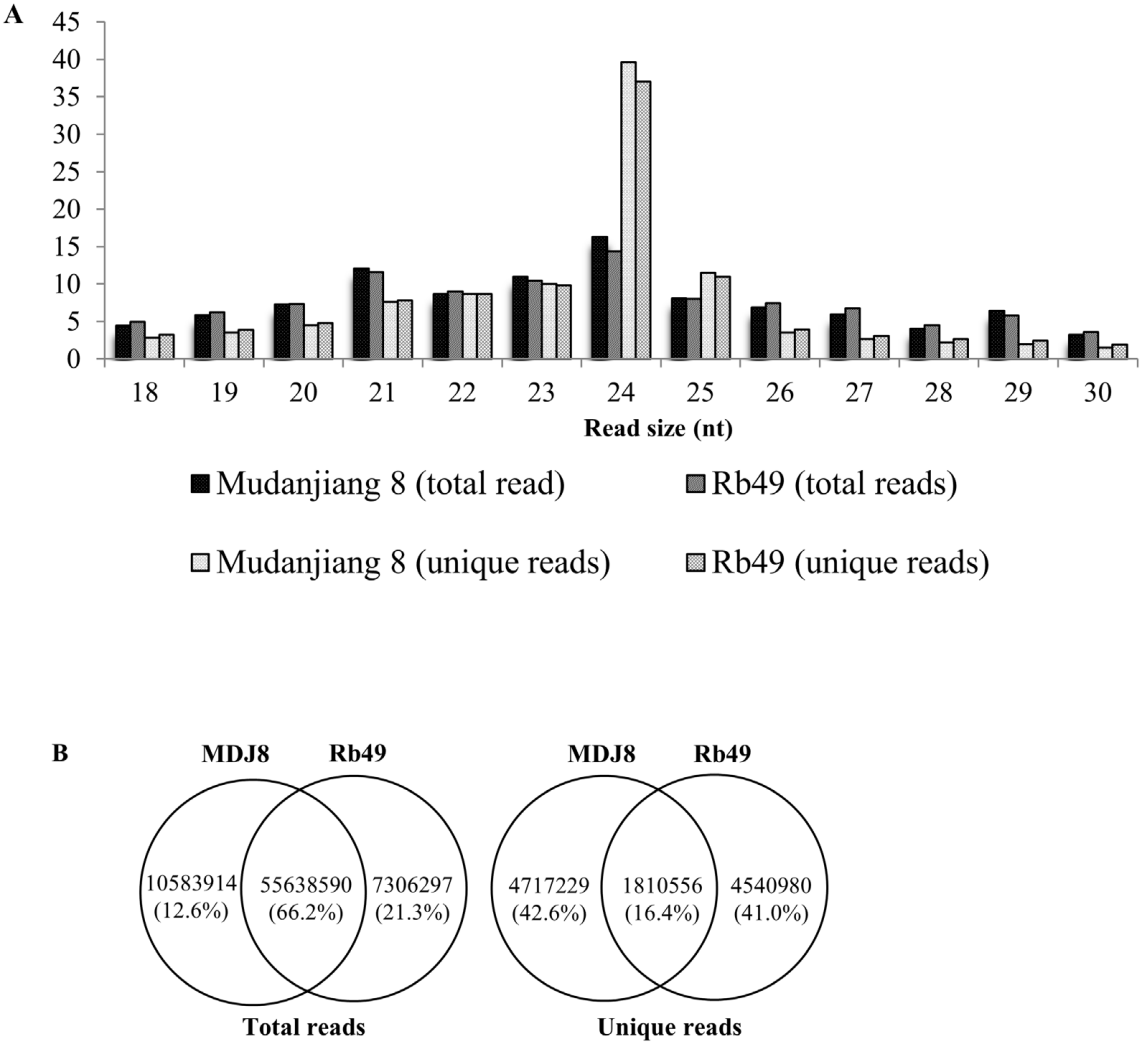
The sRNA sequencing reads from rice lines Mudanjiang 8 (MDJ8) and Rb49. A. The distribution of different sized reads. nt, nucleotide. B. Summary of the common and rice line-specific reads.

The sRNA sequencing generated a total of 66,222,504 and 73,528,801 (not including technical duplicates and biological replicates) clean reads representing 6,527,785 and 6,351,536 unique reads from rice lines MDJ8 and Rb49, respectively. The total and unique reads ranged from 18 nt to 30 nt for the 21-nt to 24-nt reads, which consisted of approximately 46.7% and 63.9% total and unique reads in both rice lines, respectively, with higher abundance than reads of other sizes (Fig 1A). This read size distribution is consistent with that of previous reports. In rice, the majority of sRNAs are 20 nt to 24 nt in length. In general, the miRNAs are 20 nt to 22 nt in length and the siRNAs are mostly 21 nt and 24 nt in length [50]. In addition, the Brassica 23-nt sRNAs may present as a new class of functional sRNAs [51]. Among the total reads, 66% were common in both rice lines, whereas among the unique reads only 16% were common in both lines (Fig 1B). This result suggests that the common reads have much higher expression levels than the rice line—specific reads. Furthermore, most of the unique reads are rice line—specific, although MDJ8 and Rb49 have the same genetic background, suggesting that these rice line—specific reads may relate with or without the function of *Xa3/Xa26*.

In total, 90.5% and 89.8% of the total reads that represented 67.8% and 65.8% of unique reads from MDJ8 and Rb49, respectively, could be perfectly (no mismatch) mapped to the rice genome (Table 1). The perfectly mapped reads consisted of various types of RNAs, including tRNA, rRNA, snoRNA, snRNA, repeat-associated, pre-miRNA, degradation tags of exons or introns, and no annotation with different abundance (Table 1). In addition, approximately 0.1% of unique reads in both rice lines were perfectly matched with the genomic sequences of *Xoo* strain PXO99^A^ (Table 1) [52]. A large fraction of the perfectly mapped unique reads (approximately 33%) was not annotated and probably includes new miRNA and siRNA candidates.

**Table 1:** Distribution of sequence reads among different categories.

Category	Mudanjiang 8		Rb49	
	Total	Unique	Total	Unique
**Mapped to genome**	59904296 (90.5%)	4425249 (67.8%)	65992944 (89.8%)	4178177 (65.8%)
tRNA	720480 (1.1%)	10270 (0.2%)	821670 (1.1%)	10369 (0.2%)
rRNA	10885520 (16.4%)	48170 (0.7%)	13752943 (18.7%)	48889 (0.8%)
snoRNA	134458 (0.2%)	4867 (0.1%)	141560 (0.2%)	5001 (0.1%)
snRNA	43247 (0.1%)	4081 (0.1%)	43341 (0.1%)	4155 (0.1%)
Repeats	22696055 (34.3%)	382314 (5.9%)	27426105 (37.3%)	359283 (5.7%)
Pre-miRNA	3010589 (4.5%)	14170 (0.2%)	2362849 (3.2%)	13285 (0.2%)
Exon	6558703 (9.9%)	1102034 (16.9%)	5835347 (7.9%)	1154937 (18.2%)
Intron	2187935 (3.3%)	581644 (8.9%)	1959682 (2.7%)	534468 (8.4%)
No-annotation	13667309 (20.6%)	2277699 (34.9%)	13649447 (18.6%)	2047790 (32.2%)
**Un-mapped**	6318208 (9.5%)	2102536 (32.2%)	7535857 (10.2%)	2173359 (34.2%)
**PXO99A**	286995 (0.4%)	6970 (0.1%)	345039 (0.5%)	5735 (0.1%)
Total	66222504 (100%)	6527785 (100%)	73528801 (100%)	6351536 (100%)

### A number of miRNAs are differentially expressed in rice response to *Xoo* infection

In total, 10,029,453 and 8,458,936 miRNA reads representing 436 and 441 unique miRNAs from MDJ8 and Rb49, respectively, and ranging from 20 nt to 24 nt in size were identified. The unique miRNAs consisted of 308 and 313 reported miRNAs in miRBase and 129 and 129 unreported miRNAs in MDJ8 and Rb49, respectively. The unreported miRNAs include novel miRNAs (named osa-miRnX; “X” indicates series number) that originated from unreported loci and new miRNAs (named osa-miRX-new) from reported pre-miRNAs, but not the known miRNAs (S4 Table). In total, 29 and 29 novel miRNAs and 100 and 100 new miRNAs were identified in rice lines MDJ8 and Rb49, respectively.

**Fig 2:**
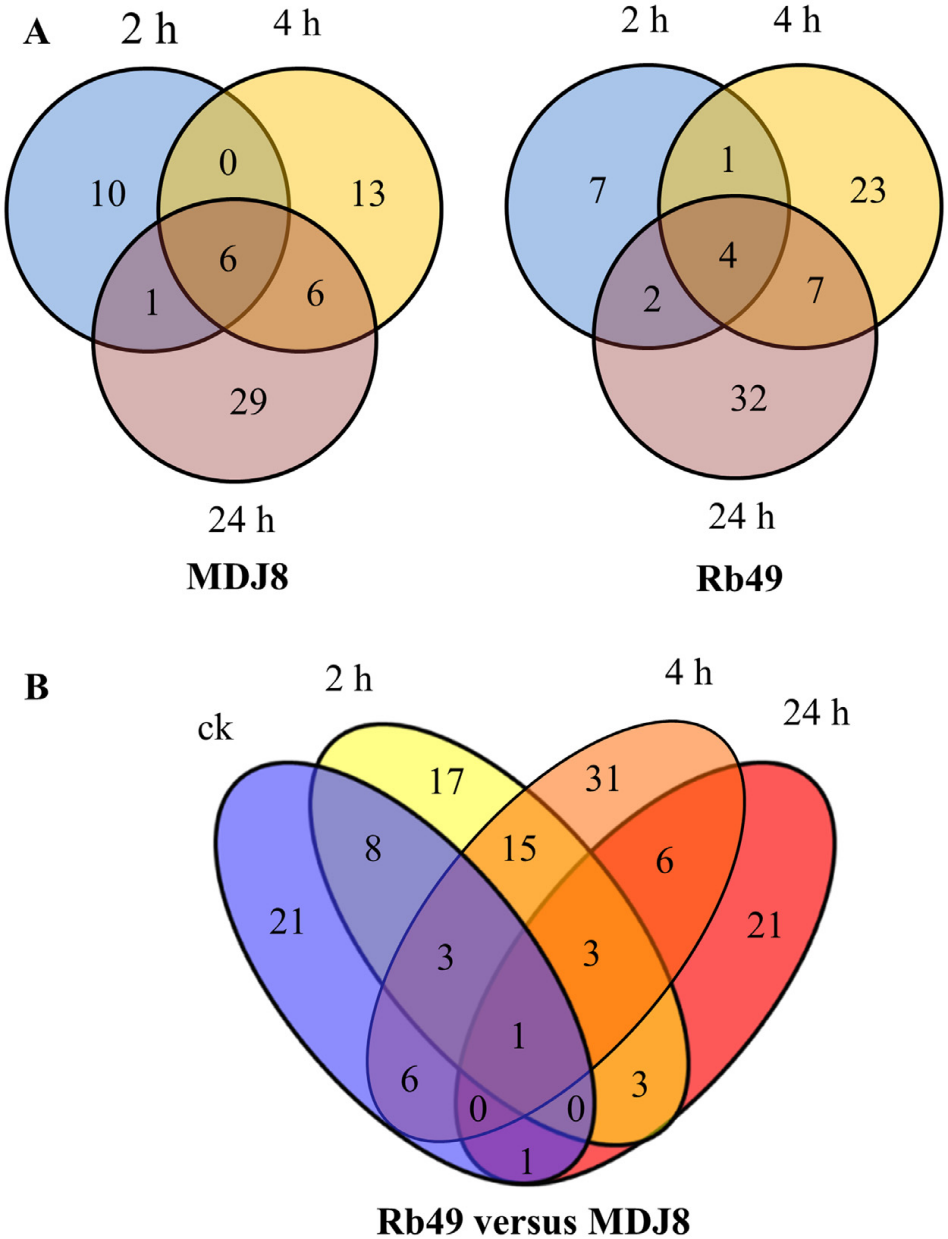
Differentially expressed miRNAs in susceptible (rice line MDJ8) and resistance (rice line Rb49) reactions. Samples were collected before inoculation (ck) and at 2, 4, and 24 h after inoculation of *Xoo*. A. Number of differentially expressed miRNAs at different times after inoculation of *Xoo* in Rb49 and MDJ8 compared with corresponding mock-inoculated plants. B. Number of differentially expressed miRNAs in Rb49 compared with MDJ8.

Compared with mock-treated samples, 65 (including 31 unreported miRNAs) and 76 (including 32 unreported miRNAs) unique miRNAs showed differential expression in rice lines MDJ8 (susceptible reaction) and Rb49 (resistant reaction), respectively, during at least one time point after *Xoo* infection (Fig 2A). In MDJ8, 41 of the 65 unique miRNAs showed induced expression, 21 showed suppressed expression, and 3 showed both induced and suppressed expression at different time points after *Xoo* infection; in Rb49, 30 of the 76 unique miRNAs showed induced expression, 36 showed suppressed expression, and 10 showed both induced and suppressed expression at different time points after *Xoo* infection (S5 Table). However, comparing the differentially expressed miRNAs between Rb49 and MDJ8, a total of 136 (including 58 unreported miRNAs) unique miRNAs were identified in Rb49 (Fig 2B; S6 Table). Among the 136 miRNAs, 19 were induced, 98 were suppressed, and 19 were both induced and suppressed at different time points after *Xoo* infection in Rb49 compared with MDJ8. This suggests that the functions of a relatively large number of genes putatively involved in *Xa3/Xa26*-mediated defense signaling may be suppressed by miRNAs without *Xoo* infection.

Among the differentially expressed miRNAs in Rb49 compared with MDJ8, several have been reported to be involved in rice—pathogen interactions. Overexpressing miR160a or miR398b enhanced rice resistance to *M*. *oryzae* by increasing the accumulation of hydrogen peroxide at the infection site and upregulating the expression of defense-related genes [19]. We found that the expression level of miR398b was significantly higher in Rb49 than in MDJ8 after *Xoo* infection, which suggests that miR398b may also be involved in rice resistance to *Xoo*. In contrast, miR160a expression decreased in Rb49 compared with MDJ8 at 2 hours after *Xoo* infection. Another study reports that miR7695 can negatively regulate an alternatively spliced transcript of Os*Nramp6*, and its overexpression in rice confers resistance to *M*. *oryzae* [18]. In the present study, miR7695 expression was barely detected. It is interesting to note that a new miRNA (miR7695-new) originated from the same precursor of miR7695, and its expression level was significantly high in Rb49 compared with MDJ8 at 24 hours after *Xoo* infection. Further study may be needed to determine whether miR7695-new is involved in rice resistance to *Xoo*.

Rb49 and MDJ8 have the same genetic background, except that Rb49 carries transgene *Xa3/Xa26* [32]. These results suggest that a large number of miRNAs are transcriptionally responsive to both the susceptible and resistant reactions, but even more miRNAs are transcriptionally responsive to *Xa3/Xa26*-mediated disease resistance. Thus, much work appears to be required to elucidate whether or which of the differentially expressed miRNAs are directly involved in *Xa3/Xa26*-mediated resistance against *Xoo*.

### Some siRNAs are also differentially expressed in rice—*Xoo* interactions

In addition to miRNA, a large number of siRNAs were also identified. Unlike miRNAs that derive from single-stranded and hairping-shaped RNA precursors, siRNAs are produced from long double-strand RNAs. Among our sequencing reads, 13,558,123 were fall into the siRNA population. After removing the reads that had multiple positions on the reference genome and did not have perfect match with the reference genome [40], 145,918 unique siRNAs with lengths ranging from 20 nt to 24 nt were identified and used for subsequent analyses (S7 Table). Strikingly, of these unique siRNAs, approximately 88% are 24-nt siRNAs, which is in accordance with a previous study of the process of rice panicle maturation [40].

**Fig 3:**
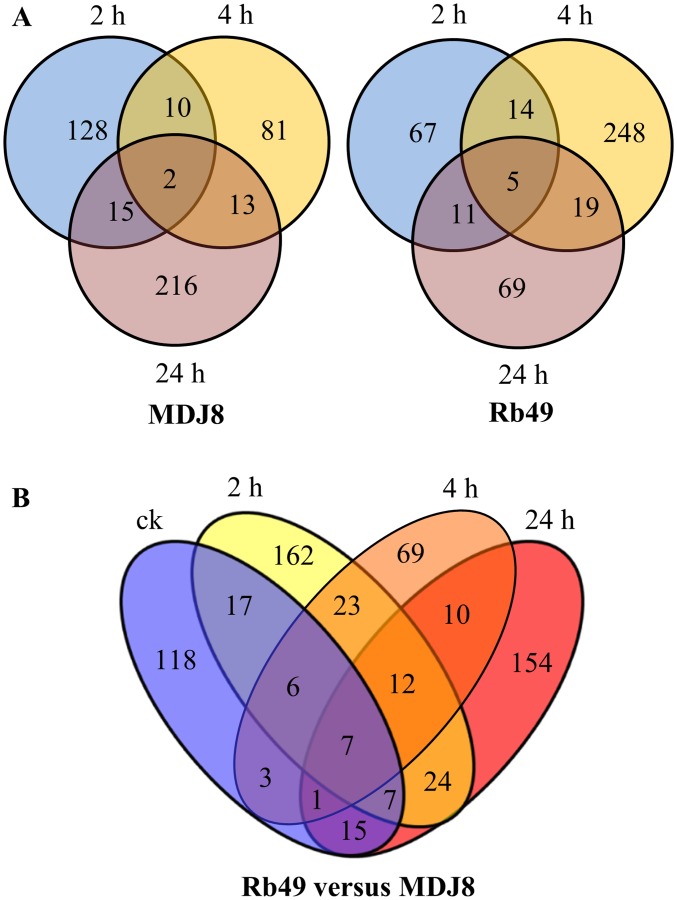
The differential expressed siRNAs in susceptible (rice line MDJ8) and resistance (rice line Rb49) reactions. Samples were collected before inoculation (ck) and at 2, 4, and 24 h after inoculation of *Xoo*. A. Numbers of differentially expressed siRNAs at different times after inoculation of *Xoo* in MDJ8 and Rb49 compared with corresponding mock-inoculated plants. B. Number of differentially expressed siRNAs in Rb49 compared with MDJ8.

Compared with mock-treated samples, 465 and 433 unique siRNAs showed differential expression in rice lines MDJ8 and Rb49, respectively, during at least one time point after *Xoo* infection (Fig 3A; S8 Table). In MDJ8, 174 of the 465 unique siRNAs showed induced expression, 273 showed suppressed expression, and 18 showed both induced and suppressed expression at different time points after *Xoo* infection; in Rb49, 81 of the 433 unique miRNAs showed induced expression, 324 showed suppressed expression, and 28 showed both induced and suppressed expression at different time points after *Xoo* infection. However, comparing the differentially expressed siRNAs between Rb49 and MDJ8, a total of 628 unique siRNAs were identified in Rb49 (Fig 3B; S9 Table). Among the 628 siRNAs, 80 were induced, 535 were suppressed, and 13 were both induced and suppressed at different time points after *Xoo* infection in Rb49 compared with MDJ8. These results suggest that, like the transcription response of miRNAs, a large number of siRNAs are also transcriptionally responsive to both the susceptible and resistant reactions, and even more siRNAs are transcriptionally responsive to *Xa3/Xa26*-mediated disease resistance. However, most of the transcriptional responsive siRNAs were suppressed in both susceptible and resistant reactions as well as in *Xa3/Xa26*-mediated resistance. Thus, siRNAs may also play an important role in reprograming the gene transcriptome in *Xa3/Xa26*-initiated defense signaling.

### A large number of genes are differentially expressed in *Xa3/Xa26*-mediated resistance

**Fig 4:**
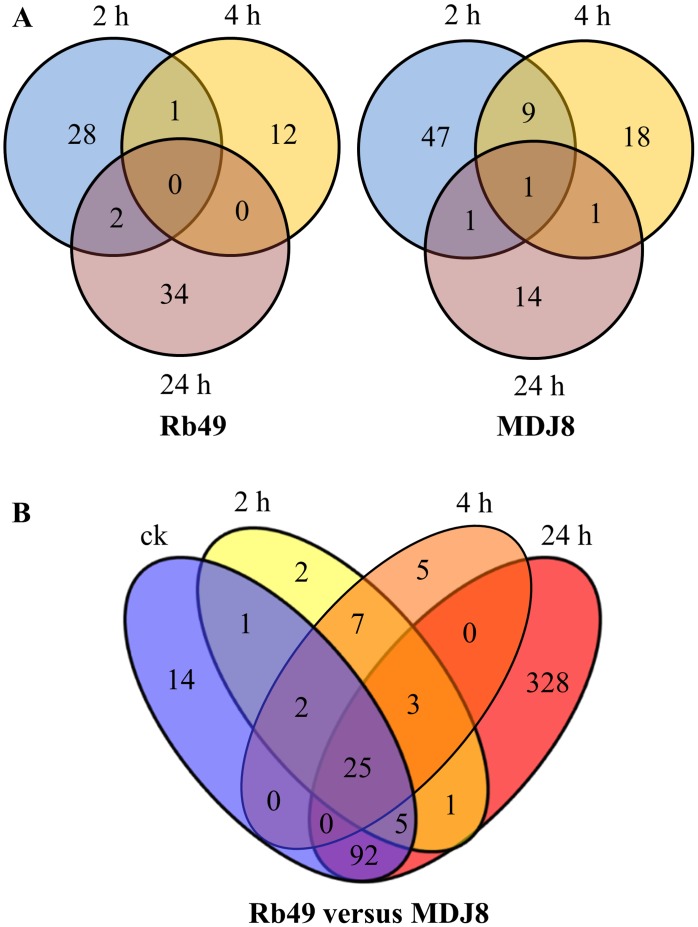
Differentially expressed genes in susceptible (rice line MDJ8) and resistance (rice line Rb49) reactions. Samples were collected before inoculation (ck) and at 2, 4, and 24 h after inoculation of *Xoo*. A. Number of differentially expressed genes at different times after inoculation of *Xoo* in Rb49 and MDJ8 compared with corresponding mock-inoculated plants. B. Number of differentially expressed genes in Rb49 compared with MDJ8.

In the 57,381 probe sets of the Affymetrix GeneChip Rice Genome Array, 33,978 probe sets, which represent 23,137 transcripts, had detected expression in the present experimental condition. Compared to the noninoculated samples, a total of 2758 and 3006 genes were differentially expressed after *Xoo* inoculation in rice lines Rb49 and MDJ8, respectively (S2A Fig). Among these genes, 1457 were differentially expressed in both rice lines, and 356 and 1549 were differentially expressed in Rb49 and MDJ8, respectively (S2B Fig). However, compared to the mock-inoculated samples, only 77 and 91 genes showed differential expression after *Xoo* inoculation in Rb49 and MDJ8, respectively (Fig 4A; S10 Table). Because the leaf clipping method was used for bacterial inoculation, a large number of differentially expressed genes detected after *Xoo* inoculation compared with noninoculated samples may be due to the wound response.

**Fig 5:**
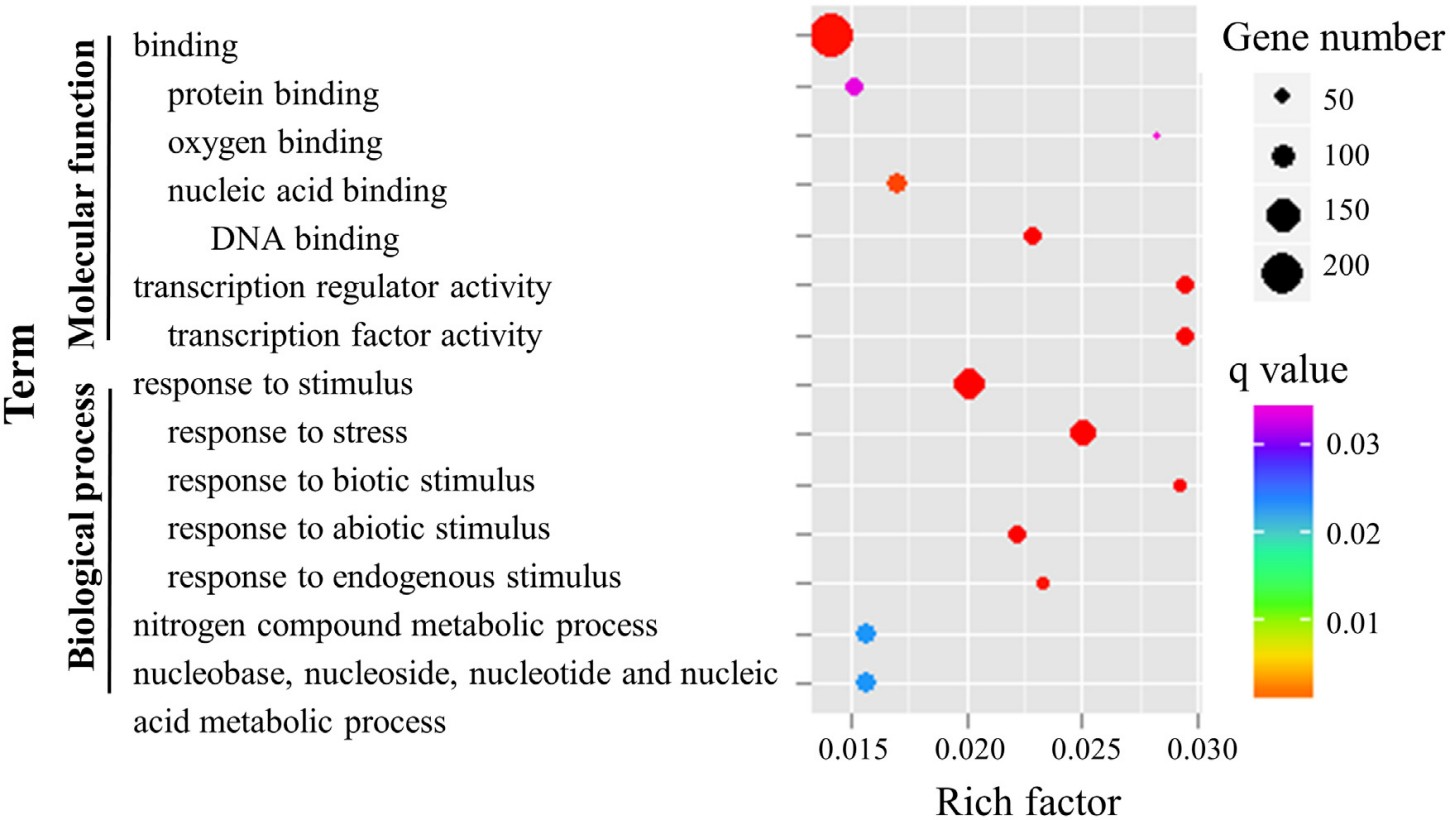
The significantly enriched GO terms in differentially expressed genes in Rb49 compared with MDJ8. The y axis stands for the name of GO term and x axis stands for rich factor. Q value means the significance of GO term. Rich factor was calculated by the number of genes mapped to the GO term divided by the number of all genes in the input list.

A total of 485 genes were differentially expressed in Rb49 compared with MDJ8 before and after *Xoo* inoculation (Fig 4B; S11 Table). Among these genes, 471 were upregulated and 14 were downregulated. A total of 383 of the 485 differentially expressed genes have annotations in the GO database. The 34,296 genes with GO annotations were used as a baseline to analyze the 383 differentially expressed genes in GO categories and subcategories, including "biological process", "cellular component", and "molecular function" ontologies. A total of 14 enriched GO terms were found to be enriched in "biological process" and "molecular function" ontologies, and no term in "cellular component" ontology was found to be enriched (Fig 5; S12 Table), indicating that the genes in these terms may be associated with the resistant response in comparison with the susceptible response.

We also separately classified the upregulated and downregulated genes. Among the upregulated genes, there were four significantly enriched terms: "response to stimulus" (GO:0050896) and "nitrogen compound metabolic process" (GO:0006807) in the "biological process", and "binding" (GO:0005488) and "transcription regulator activity" (GO:0030528) in the "molecular function". This classification suggests that in response to *Xoo* infection, nitrogen compound metabolism was rapidly changed and transcription factors (15 WRKYs and 9 MYBs) were activated to facilitate *Xa3/Xa26*-mediated resistance. Among the 14 downregulated genes (nine with GO annotations), only one subcategory (GO:0016491, oxidoreductase activity) in the "molecular function" was found to be significantly enriched, suggesting that suppressing oxidoreductase activity may be important in early defense signaling initiated by *Xa3/Xa26*.

### Expression of a group of genes putatively response to *Xa3/Xa26*-mediated resistance is likely regulated by sRNAs

To study the relationship between miRNAs and their target genes, we integrated the differentially expressed miRNA data with their predicted target gene data. Each of the 136 differentially expressed unique miRNAs in Rb49 compared with MDJ8 targeted 1 to 23 candidate genes. A total of 694 candidate genes were identified (S13 Table). There were 308 miRNA—gene pairs with PCC value small than -0.001 in expression level. Among the 308 pairs, the expression of 39 pairs was negatively correlated with the PCC values ranging from -0.502 to -0.814. The homologs of some of the genes in the 39 pairs have been reported to be involved in plant—pathogen interactions. For example, the expression of osa-miR397a-new and LOC_Os01g62480 encoding a putative laccase precursor protein was negatively correlated (PCC -0.553). The laccase gene underlies part of the dual resistance QTL against soybean cyst nematode [53]. The expression of osa-miR1430-new negatively correlated (PCC -0.557) with the expression of LOC_Os01g04409 encoding an OsWAK receptor—like kinase. WAK-like proteins have been reported to regulate disease resistance in Arabidopsis, rice, and maize [54–56]. The expression of osa-miR2864.1 and LOC_Os03g55040 encoding a UDP-glucosyltransferase domain—containing protein negatively correlated (PCC -0.502). A UDP-glucosyltransferase is required for Arabidopsis nonhost resistance to rust pathogen [57]. These results suggest that further study is needed to find the relationships of the 39 pairs of miRNAs and their potential target genes in rice resistance to *Xoo*.

**Fig 6:**
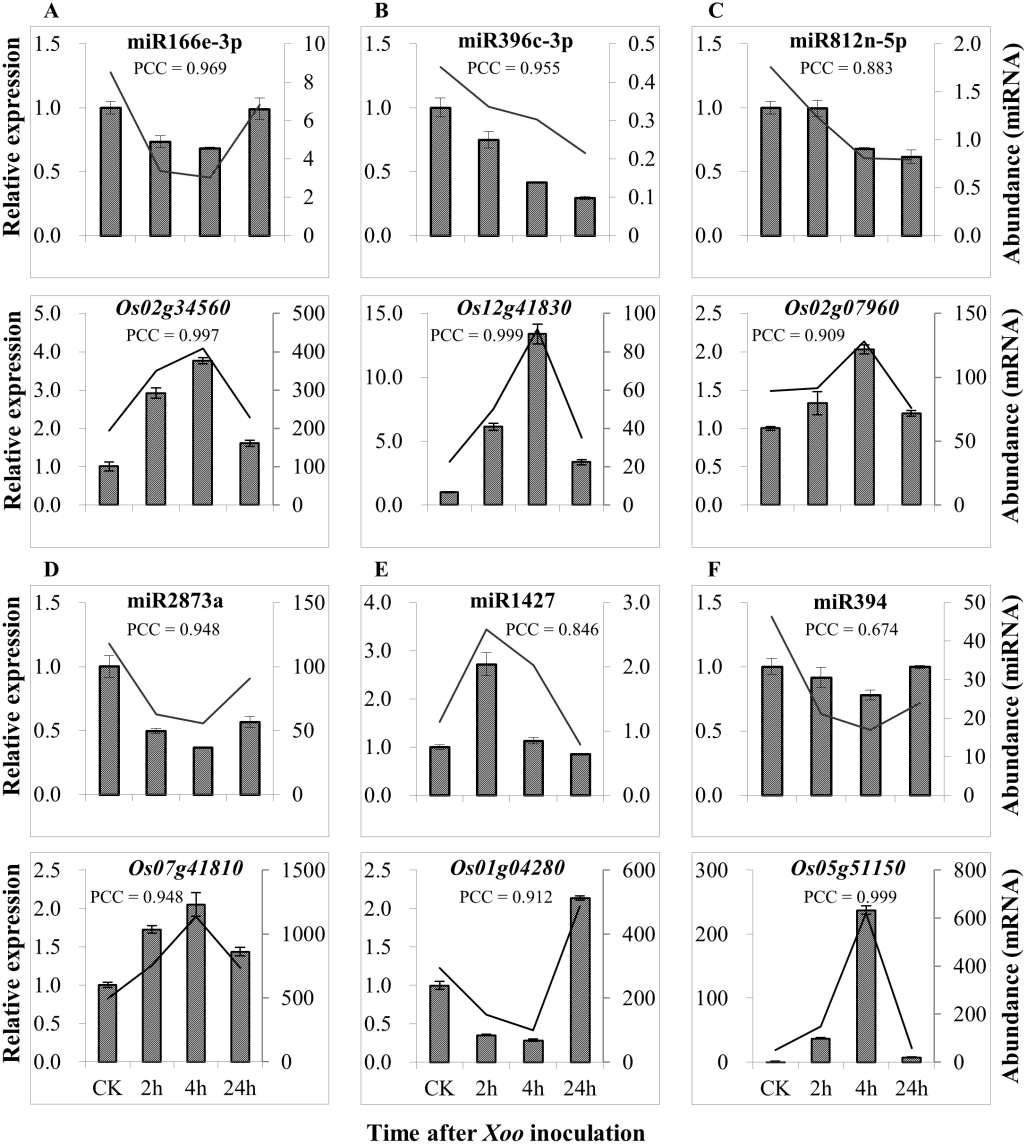
The expression of genes was negatively associated with the expression of miRNAs. Bars (left y axis) represents the expression detected by qRT-PCR, while lines (right y axis) represent the normalized expression detected by Solexa sequencing or the expression by the Genechip analysis. Each bar represents mean (three replicates) ± standard deviation. A, B, C, D, E and F, different pairs of miRNA and gene. The miRNAs were differentially expressed in *Xoo*-inoculated Rb49 compared with mock-inoculated Rb49 (S5 Table). PCC, Pearson’s correlation coefficient. CK, before *Xoo* inoculation.

To further examine the prediction of the relationship of miRNAs and their putatively regulated genes, we analyzed six predicted miRNA—gene pairs in which miRNAs were differentially expressed in Rb49 after *Xoo* inoculation compared with mock-inoculated plants (S5 Table) by qRT-PCR. The expression patterns of both miRNAs and genes detected by qRT-PCR correlated with the expression patterns of miRNAs and genes analyzed by Solexa sequencing and GeneChip analysis, respectively (Fig 6). In addition, the expression levels of miRNAs were negatively associated with the expression levels of their potential target genes. When the expression of miRNAs was suppressed after *Xoo* infection compared with control (ck), the expression of their potential target genes was induced. In contrast, when the expression of miRNA was induced after *Xoo* infection compared with control, the expression of its potential target gene was suppressed (Fig 6). These results further suggest that our GeneChip data and sequencing data are reliable and that the predicted transcriptional negatively associated miRNA—gene pairs can be candidates for studying rice—*Xoo* interactions.

**Fig 7:**
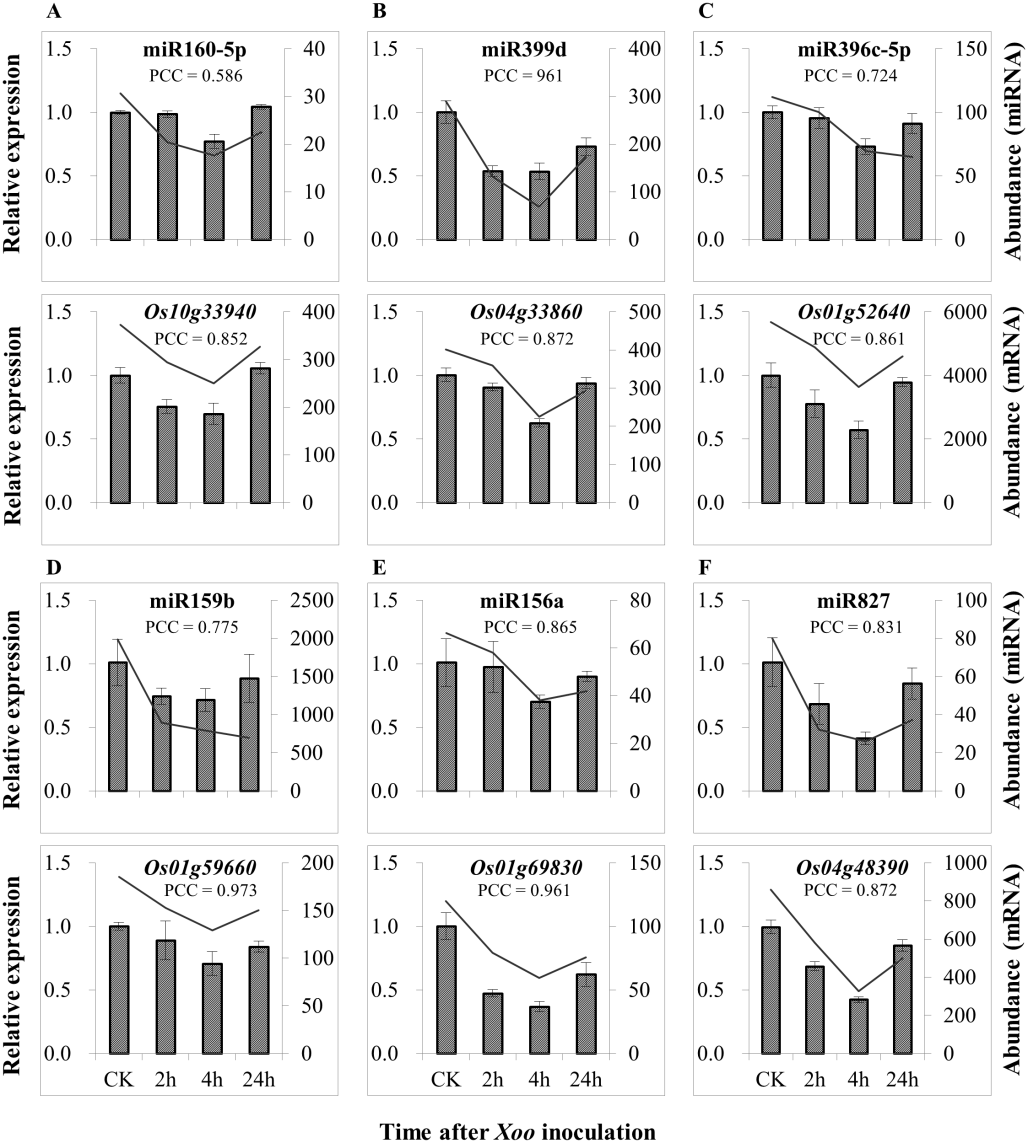
The expression of genes was associated with the expression of miRNAs. Bars (left y axis) represents the expression detected by qRT-PCR, while lines (right y axis) represent the normalized expression detected by Solexa sequencing or the expression by the Genechip analysis. Each bar represents mean (three replicates) ± standard deviation. A, B, C, D, E and F, different pairs of miRNA and gene. The miRNAs were differentially expressed in *Xoo*-inoculated Rb49 compared with mock-inoculated Rb49 (S5 Table). PCC, Pearson’s correlation coefficient. CK, before *Xoo* inoculation.

The expressions of most of the differentially expressed miRNAs and their predicted target genes had large PCC values (S13 Table). Some of the pairs were even positively correlated in expression level. We re-examined six predicted miRNA—gene pairs which expression was positively correlated (S13 Table). Each of miRNA-gene pairs detected by qRT-PCR showed similar expression pattern in response to *Xoo* infection, which was consistent with the expression patterns of miRNAs and genes analyzed by Solexa sequencing and GeneChip analysis, respectively (Fig 7; S13 Table). This phenomenon may be due to a similar feedback regulation between miRNAs and candidate target genes as the co-expression of ath-miR164a and *CUC2* genes in Arabidopsis [58]. Another possibility is that some miRNAs can repress translation, as the case that ath-miR167 translationally represses *ARF6* in Arabidopsis [59]. Most of these sRNAs did not exhibit a significantly negative correlation with their predicted targets regarding expression levels, suggesting that a translational repression mechanism may play an important role in *Xa3/Xa26*-mediated resistance.

## Conclusion

*Xa3/Xa26* confers durable and qualitative resistance to *Xoo*. The present results suggest that both miRNAs and siRNAs may be involved in the regulation of *Xa3/Xa26*-meidated resistance. The simultaneous genome-wide analyses of the expression of sRNAs and genes will facilitate further studies of the roles of sRNAs and their target genes in *Xa3/Xa26*-initiated defense signaling.

## Supporting Information

S1 Fig Correlation of miRNA abundance between two technical duplicates (A) and biological replicates (B). Data are from Illumina high-throughput deep sequencing. Rb49-4h-mock, sample from rice line Rb49 at 4 hours after mock inoculation; MDJ8-ck, sample from rice line Mudanjiang 8 before inoculation. r, correlation coefficient.
(PDF)

S2 Fig Differentially expressed genes at different time points after inoculation of *Xoo* compared with noninoculated samples in rice lines Rb49 and Mudanjiang 8 (MDJ8). A. The number of differentially expressed genes at different time points. B. The number of differentially expressed genes in Rb49 and MDJ8 during all three time points (total of 3452 genes).
(PDF)

S1 Table Primers used for quantitative polymerase chain reaction in miRNA expression analysis. (PDF)

S2 Table Primers used for quantitative polymerase chain reaction in gene expression analysis. (PDF)

S3 Table Correlation coefficients of two biological replicates for gene chip analysis. (PDF)

S4 Table Normalized reads of rice miRNAs before and after PXO61 inoculation in rice lines Rb49 and Mudanjiang 8 (MDJ8). (XLS)

S5 Table Differentially expressed miRNA in rice lines Mudanjiang 8 (MDJ8) and Rb49 after *Xoo* inoculation during at least one time point compared with corresponding mock-inoculated plants. (XLS)

S6 Table Ratio of the normalized miRNA sequence reads of the miRNAs between Rb49 and Mudanjiang 8 (MDJ8) after PXO61 inoculation. (XLS)

S7 Table Normalized reads of rice siRNAs before and after PXO61 inoculation in rice lines Mudanjiang 8 (MDJ8) and Rb49. (XLS)

S8 Table Ratio of the normalized siRNA sequence reads between PXO61 inoculation and mock treatments in rice lines Rb49 and Mudanjiang 8 (MDJ8). (XLS)

S9 Table Ratio of the normalized siRNA sequence reads between Rb49 and Mudanjiang 8 (MDJ8) after PXO61 inoculation. (XLS)

S10 Table Ratio of the differentially expressed genes between *Xoo*-inoculated and mock-inoculated samples in rice lines Rb49 and Mudanjiang 8 (MDJ8). (XLS)

S11 Table Ratio of the differentially expressed genes between Rb49 and Mudanjiang 8 (MDJ8) before and after *Xoo* inoculation. (XLS)

S12 Table Functional classification of differentially expressed genes between Rb49 and Mudanjiang 8 (MDJ8) based on Gene Ontology (GO). (XLS)

S13 Table Correlation of differentially expressed miRNA in Rb49 (compared with Mudanjiang 8 [MDJ8]) and its predicted target using Pearson’s coefficient. (XLS)
