# Supplementary material for: Small RNAs and Gene Network in a Durable Disease Resistance Gene—Mediated Defense Responses in Rice
Source: PLoS One. 2015 Sep 3;10(9):e0137360. doi: 10.1371/journal.pone.0137360 (PMC4559425; doi:10.1371/journal.pone.0137360)
Supplement: S1 Fig — Data are from Illumina high-throughput deep sequencing. Rb49-4h-mock, sample from rice line Rb49 at 4 hours after mock inoculation; MDJ8-ck, sample from rice line Mudanjiang 8 before inoculation. r, correlation coefficient. (PDF) [file pone.0137360.s001.pdf]

A

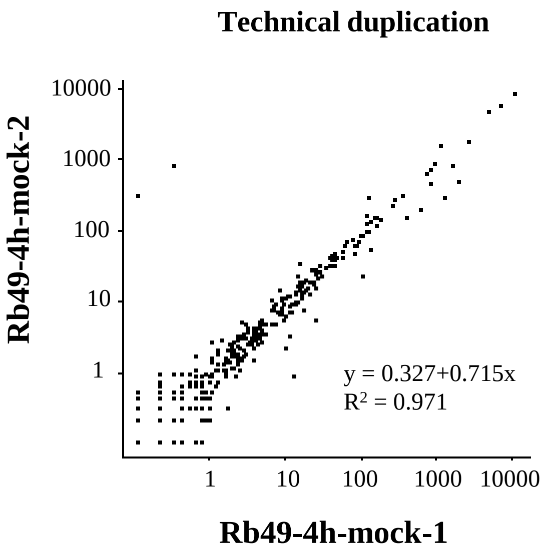

B

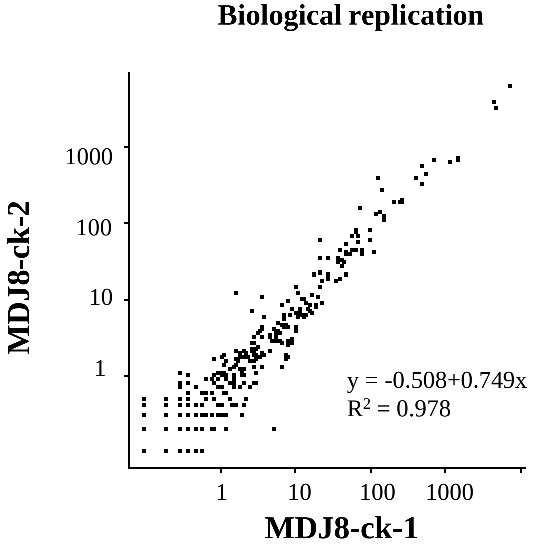

S1 Fig. Correlation of miRNA abundance between two technical duplicates (A) and biological replicates (B). Data are from Illumina high-throughput deep sequencing. Rb49-4h-mock, sample from rice line Rb49 at 4 hours after mock inoculation; MDJ8-ck, sample from rice line Mudanjiang 8 before inoculation. r, correlation coefficient.
