# Supplementary material for: Small RNAs and Gene Network in a Durable Disease Resistance Gene—Mediated Defense Responses in Rice
Source: PLoS One. 2015 Sep 3;10(9):e0137360. doi: 10.1371/journal.pone.0137360 (PMC4559425; doi:10.1371/journal.pone.0137360)
Supplement: S2 Fig — A. The number of differentially expressed genes at different time points. B. The number of differentially expressed genes in Rb49 and MDJ8 during all three time points (total of 3452 genes). (PDF) [file pone.0137360.s002.pdf]

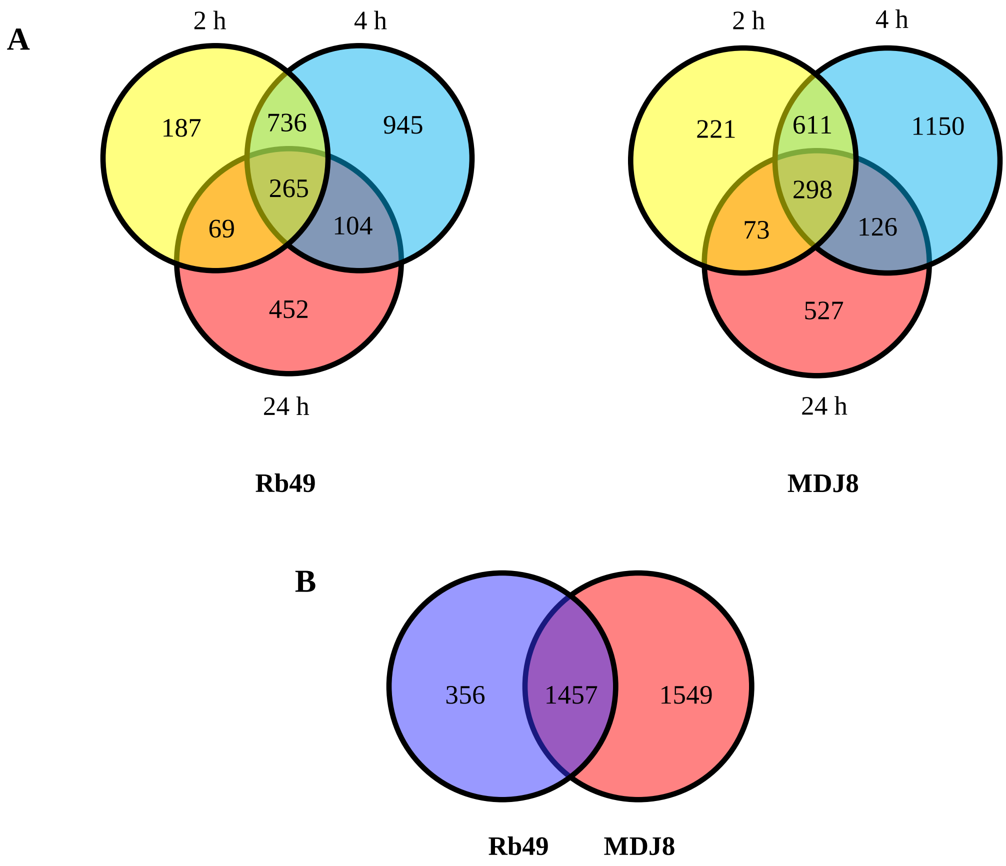

S2 Fig. Differentially expressed genes at different time points after inoculation of *Xoo* compared with noninoculated samples in rice lines Rb49 and Mudanjiang 8 (MDJ8). A. The number of differentially expressed genes at different time points. B. The number of differentially expressed genes in Rb49 and MDJ8 during all three time points (total of 3452 genes).
