# Supplementary material for: Small RNAs and Gene Network in a Durable Disease Resistance Gene—Mediated Defense Responses in Rice
Source: PLoS One. 2015 Sep 3;10(9):e0137360. doi: 10.1371/journal.pone.0137360 (PMC4559425; doi:10.1371/journal.pone.0137360)
Supplement: S1 Table — (PDF) [file pone.0137360.s003.pdf]

S1 Table. Primers used for quantitative polymerase chain reaction in miRNA expression analysis

miRNA[a]	Stem-loop primer	Forward primer 5'-3'
miR166e-3p	GTCGTATCCAGTGCAGGGTCCGAGGTATTCGCACTGGATACGACggggaat	gcggcggTCGAACCAGGCTTCA
miR396c-3p	GTCGTATCCAGTGCAGGGTCCGAGGTATTCGCACTGGATACGACcttccc	gcggcggGGTCAAGAAAGCTGT
miR812n-5p	GTCGTATCCAGTGCAGGGTCCGAGGTATTCGCACTGGATACGACgcacgg	gcggcggAAGTGCAGCCATGAGTTT
miR2873a	GTCGTATCCAGTGCAGGGTCCGAGGTATTCGCACTGGATACGACgttacc	gcggcggAAGTTTGGACTTAAATTT
miR1427	GTCGTATCCAGTGCAGGGTCCGAGGTATTCGCACTGGATACGACgcgcca	gcggcggTGCGGAACCGTGCGG
miR394	GTCGTATCCAGTGCAGGGTCCGAGGTATTCGCACTGGATACGACggaggt	gcggcggTTGGCATTCTGTCC
miR399d	GTCGTATCCAGTGCAGGGTCCGAGGTATTCGCACTGGATACGACcagggc	gatccTGCCAAAGGAGAGTT
miR396c-5p	GTCGTATCCAGTGCAGGGTCCGAGGTATTCGCACTGGATACGACaagttc	gatccTTCCACAGCTTTCTT
miR159b	GTCGTATCCAGTGCAGGGTCCGAGGTATTCGCACTGGATACGACcagagc	gatccTTTGGATTGAAGGGA
miR156a	GTCGTATCCAGTGCAGGGTCCGAGGTATTCGCACTGGATACGACgtgctc	gatccTGACAGAAGAGAGT
miR827	GTCGTATCCAGTGCAGGGTCCGAGGTATTCGCACTGGATACGACtgtttg	gatccTTAGATGACCATCAG
miR160a-5p	GTCGTATCCAGTGCAGGGTCCGAGGTATTCGCACTGGATACGACtggcat	gatccTGCCTGGCTCCCTGT
Universal reverse primer	CCAGTGCAGGGTCCGAGGT	

[a]The miRNA ID in the microRNA database (http://www.mirbase.org/), version 21.
