# Supplementary material for: Small RNAs and Gene Network in a Durable Disease Resistance Gene—Mediated Defense Responses in Rice
Source: PLoS One. 2015 Sep 3;10(9):e0137360. doi: 10.1371/journal.pone.0137360 (PMC4559425; doi:10.1371/journal.pone.0137360)
Supplement: S2 Table — (PDF) [file pone.0137360.s004.pdf]

S2 Table. Primers used for quantitative polymerase chain reaction in gene expression analysis

Gene (RGAP code[a] or GenBank accession number)	Primer name	Forward primer (5'-3')	Reverse primer (5'-3')	Product size (nt)
LOC_Os02g34560	560realF/R	CTTGGTCTATCGCAGGGTATTT	AGGCTTCATTGCCTTATCCTC	98
LOC_Os12g41830	830realF/R	GGAAAGCTCATGGGTGTGATTA	GTCCATCTTTGCCTCCATCAA	121
LOC_Os02g07960	960realF/R	GTGCAGAATATGGTCAGAGGTT	GGCGAGCATTCCATGATAGT	114
LOC_Os07g41810	810realF/R	CTCTCAGCTTGATGACCATTCT	CAAGGTATGAACTCTGCCTCTC	139
LOC_Os01g04280	280realF/R	CGTCTACGTCAACTCCATCTTC	GGCCTCTTGGAGGTTGTTT	148
LOC_Os05g51150	150realF/R	GTGACTCCGGACACATCTTTAG	CAGTACGTGCCCTTCTCTTG	114
LOC_Os04g33860	33860realF/R	CTGCTTCATCTACGTCATGCT	TGGTCTTCCTGCTGCTATTG	131
LOC_Os01g52640	52640realF/R	GCCATATAGCATCTAGCGACTT	GCCTCTCATGTTCCCTCATT	93
LOC_Os01g59660	59660realF/R	ACCCTAAGATCCGGGAAGAA	TCTGGGCTAAGAACCGTAGTA	92
LOC_Os01g69830	69830realF/R	CAAGTGCACAGCTGCAATAC	CTACCACGATGAGAAAGGAAGAG	104
LOC_Os04g48390	48390realF/R	TTGCAGACGCGACGATTAC	GAGGCTATGCAGATGACAAGAG	94
LOC_Os10g33940	33940realF/R	CCCTGGAGTTACTGGAAATAGC	TCGTGACCTTGAGATGAGAAAC	93
Actin (X15865)	Actin-F/R	TGTATGCCAGTGGTCGTACCA	CCAGCAAGGTCGAGACGAA	121

[a]RGAP (Rice Genome Annotation Project, http://rice.plantbiology.msu.edu/).
