# Supplementary material for: Small RNAs and Gene Network in a Durable Disease Resistance Gene—Mediated Defense Responses in Rice
Source: PLoS One. 2015 Sep 3;10(9):e0137360. doi: 10.1371/journal.pone.0137360 (PMC4559425; doi:10.1371/journal.pone.0137360)
Supplement: S3 Table — (PDF) [file pone.0137360.s005.pdf]

S3 Table. Correlation coefficients of two biological replicates for gene chip analysis

Rice variety	Mudanjiang 8							Rb49						
Treatment	ck	PXO61			mock			ck	PXO61			mock		
		2h	4h	1d	2h	4h	1d		2h	4h	1d	2h	4h	1d
Correlation	0.994	0.997	0.997	0.997	0.996	0.998	0.994	0.995	0.997	0.995	0.996	0.993	0.995	0.995
